# Oxidative Stress‐Induced Expression Levels of *PXDN* and *NF*‐*κB* in Type 2 Diabetic Patients With Nephropathy

**DOI:** 10.1002/edm2.70252

**Published:** 2026-06-12

**Authors:** Zakira Hanin, Aisha Anees Ahmed, Shuhd Salem Al Nahdi, Sumayya Althaf Kasim, Mahir Khalil Jallo, Rajaram Rhambau Jagdale, Dalia A. Gaber

**Affiliations:** ^1^ Bachelor of Biomedical Science Gulf Medical University Ajman UAE; ^2^ Department of Clinical Sciences, College of Medicine Gulf Medical University Ajman UAE; ^3^ Center of Endocrinology Thumbay University Hospital‐ Ajman Ajman UAE; ^4^ Department of Biomedical Sciences, College of Medicine Gulf Medical University Ajman UAE

**Keywords:** biomarkers, diabetic nephropathy, MDA, *NF‐κB*, oxidative stress, *PXDN*

## Abstract

**Background:**

Diabetic nephropathy (DN) is the leading cause of end‐stage renal disease in patients with diabetes mellitus (DM). Early detection remains difficult, but essential for timely management. In this study, we assessed Peroxidasin (*PXDN*) gene expression as a biomarker for early detection of DN and for reflection of oxidative stress status. We also compared *PXDN* and Nuclear factor‐κB (*NF‐κB)* expression levels in DN patients, aiming to identify a reliable diagnostic biomarker.

**Methods:**

This prospective cross‐sectional study included 40 patients with T2DM with and without nephropathy, and 20 matched healthy controls. *PXDN* mRNA expression was quantified by real‐time polymerase chain reaction (qRT‐PCR). Biochemical analyses of malondialdehyde (MDA) and total antioxidant capacity (TAC) were also done to assess the oxidative stress status. Laboratory parameters, including glycated haemoglobin, urinary albumin‐to‐creatinine ratio, estimated glomerular filtration rate, and microalbuminuria, were measured and correlated with gene expression levels and the oxidative stress status.

**Results:**

*PXDN* mRNA expression was significantly upregulated in patients with DN compared to diabetic cases without nephropathy and control subjects. MDA levels were significantly elevated in both diabetic groups relative to controls, but no significant difference was found between DN and DM without renal impairment. TAC was significantly lower in DN cases compared to DM and controls. The *NF‐κB* gene was upregulated in both diabetic groups compared to control subjects, but no significant difference was found between DN and DM without nephropathy. Furthermore, *PXDN* expression did not correlate with MDA, TAC, or *NF‐κB* levels. Receiver operating characteristic (ROC) analysis identified *PXDN* as a highly specific and sensitive marker for DN, outperforming conventional oxidative stress biomarkers in distinguishing DN from both diabetic and healthy individuals.

**Conclusion:**

*PXDN* is significantly upregulated in DN and may serve as a sensitive and specific molecular marker for early detection of nephropathy compared to *NF‐κB*.

## Graphical abstract

PXDN expression is markedly upregulated in diabetic nephropathy and, versus NF‐κB and oxidative stress markers, shows superior diagnostic performance (AUC 0.79; 90% specificity), supporting PXDN as a sensitive, specific non‐invasive biomarker for early DN.

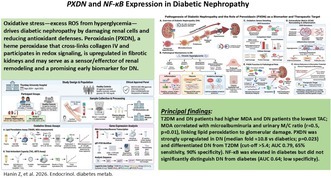

## 1 Introduction

Diabetic nephropathy (DN) is one of the most prevalent and serious microvascular complications of type 2 diabetes mellitus (T2DM), affecting approximately 20%–40% of individuals with diabetes and serving as the leading cause of chronic kidney disease (CKD) and end‐stage renal disease (ESRD) globally [1, 2]. As the global incidence of T2DM rises, so too does the burden of DN, posing significant challenges for healthcare systems and patients alike.

The prevalence of T2DM in the Emirati population is estimated to be 53.43%. Based on eGFR, 24.25% of patients have values of 60–89 mL/min/1.73 m^2^, while 7.74% of patients have eGFR < 60 mL/min/1.73 m^2^, indicating elevated renal risk [1].

The pathogenesis of DN is multifactorial, involving chronic hyperglycemia‐induced metabolic and hemodynamic disturbances that progressively impair renal structure and function. These disturbances include the activation of several damaging pathways, such as the polyol pathway, protein kinase C signalling, hexosamine biosynthesis, and the formation of advanced glycation end products (AGEs), which collectively promote inflammation, fibrosis, and oxidative damage in kidney tissues [2, 3]. The clinical progression of DN typically begins with glomerular hyperfiltration and microalbuminuria, eventually advancing to overt proteinuria, declining estimated glomerular filtration rate (eGFR), hypertension, and ultimately ESRD [1, 2].

Oxidative stress plays a central role in the initiation and progression of DN. Defined as an imbalance between the production of reactive oxygen species (ROS) and the antioxidant defence system, oxidative stress causes damage to cellular lipids, proteins, and nucleic acids, disrupting cellular integrity and signalling pathways [2, 4]. Hyperglycemia exacerbates oxidative stress via multiple mechanisms, including mitochondrial dysfunction, NADPH oxidase activation, and impaired redox homeostasis [3, 5]. Renal cells, particularly podocytes and tubular epithelial cells, are especially vulnerable to ROS due to their high metabolic activity and exposure to filtered glucose and inflammatory mediators [4]. Numerous studies have shown elevated levels of oxidative stress biomarkers in patients with DN compared to those with diabetes alone or healthy controls. Malondialdehyde (MDA), a marker of lipid peroxidation, is significantly increased in DN and reflects ROS‐induced cellular damage [1, 6]. Simultaneously, antioxidant enzyme activities such as superoxide dismutase (SOD), glutathione peroxidase (GPx), and catalase are often diminished in DN, further aggravating oxidative injury [3]. These findings underscore the pathophysiological importance of oxidative stress as both a driver and a marker of renal damage.

Given the limitations of conventional markers like serum creatinine and urinary albumin in detecting early renal injury—particularly in patients with normoalbuminuric diabetic nephropathy—there is a growing need to identify novel biomarkers that reflect upstream pathological changes such as oxidative stress, ECM remodelling, and inflammation [3, 5].

Extensive experimental data highlighted chronic inflammation driven by NF‐κB activation as a central contributor to DN. Stimulated by factors such as hyperglycemia, NF‐κB induces numerous proinflammatory cytokines, chemokines, and adhesion molecules, producing DN hallmarks: podocyte damage, excessive extracellular matrix deposition, glomerulosclerosis, epithelial–mesenchymal transition, tubular atrophy, and increased proteinuria. Thus, NF‐κB represents a compelling therapeutic target for DN [7].

Peroxidasin (*PXDN*) is an emerging candidate in this context. *PXDN* is a multifunctional, heme‐containing peroxidase secreted into the extracellular matrix (ECM), where it facilitates sulfilimine bond formation between collagen IV protomers, a process crucial for maintaining basement membrane integrity [6]. *PXDN's* dual enzymatic and structural roles allow it to participate in ECM stabilization, redox signalling, and tissue remodelling [8]. It is broadly expressed in multiple tissues, including the kidneys, and is particularly enriched in areas undergoing active remodelling, such as in fibrotic or inflamed tissues [6, 9]. In the diabetic kidney, the ECM undergoes significant alterations due to hyperglycemia‐induced oxidative stress and inflammatory responses. These changes lead to basement membrane thickening, mesangial expansion, and interstitial fibrosis—hallmarks of DN [10, 11]. Given the subtle onset and irreversible nature of late‐stage DN, early identification and intervention remain critical challenges in clinical practice. This highlights the necessity of advancing our understanding of DN pathogenesis and suggests early biomarkers that reflect kidney damage [12].

*PXDN* is responsive to these conditions and may be upregulated in response to oxidative cues, potentially acting as both a sensor and effector of redox and matrix homeostasis [6, 12]. Studies have shown that *PXDN* is incorporated into the ECM of fibrotic kidneys and that its dysregulation can disrupt matrix organization and promote fibrosis [8, 9].

Despite its promising role, the diagnostic role of *PXDN* in diabetic nephropathy remains underexplored. Its expression patterns in diabetes and diabetes with nephropathy have only recently begun to be investigated. Given that *PXDN* links oxidative stress to ECM remodelling, two major pathological processes in DN, it holds potential as a sensitive biomarker and a target for early intervention [6, 9]. PXDN may be a potential therapeutic target for this condition. However, its specific role in the context of diabetic nephropathy and its relationship with oxidative stress‐induced injury have not been extensively explored.

This study aims to assess and compare oxidative stress in T2DM patients with and without nephropathy, and to analyse the differential *PXDN* gene expression between diabetic groups to assess its potential use as an early marker of renal damage.

## 2 Patients and Methods

### 2.1 Study Design

This prospective cross‐sectional study was conducted among patients with T2DM attending the Thumbay University Hospital endocrinology and nephrology outpatient clinics between September 2024 and April 2025.

### 2.2 Study Population

A total of 60 participants contributed to this study. Twenty‐nine males and 11 females with type 2 diabetes mellitus, in addition to 20 age‐ and sex‐matched healthy controls with random blood glucose levels between 70 and 140 mg/dL. All the participants were provided with comprehensive study information before obtaining informed written consent.

Sample size was calculated using MedCalc version 12.6 (MedCalc Software, Mariakerke, Belgium). Based on a previous study [13], which measured oxidative stress in T2DM patients, the mean ± SD values of MDA were: 35.75 ± 0.53 nmol/mL in controls, 38.29 ± 0.41 nmol/mL in T2DM patients, and 49.39 ± 0.78 nmol/mL in those with diabetic nephropathy. The small standard deviations suggested a low required sample size for statistical significance.

#### 2.2.1 Inclusion Criteria for T2DM

- Adults aged 40 –70 years with a confirmed diagnosis of T2DM
- This cohort was divided into two groups: Group 1, which included patients with DN
  (determined by a urinary albumin: creatinine ratio of 30–300 mg/g, microalbuminuria (30–300 mg/day), or eGFR < 60 mL/min/1.73 m^2^), and Group 2, which included patients with T2DM of ≤ 2 years' duration and HbA1c ≥ 6.5%, without clinical or laboratory evidence of nephropathy.

#### 2.2.2 Exclusion Criteria

Presence of non‐diabetic kidney disease or other chronic renal pathology.

### 2.3 Study Setting

Ten mL of venous blood samples were collected in EDTA sterile vacutainers from patients and control subjects. A certified phlebotomist assisted in blood sampling at TUH. Blood samples were aliquoted for subsequent biochemical and molecular analyses. All laboratory procedures were conducted at TUH, at the Biochemistry laboratory, and at Thumbay Research Institute for Precision Medicine (TRIPM), Gulf Medical University.

### 2.4 Ethical Considerations

This study was conducted following strict ethical standards to ensure the protection of participant rights and confidentiality. Ethical approval for the study was obtained from both the Ministry of Health and Prevention (MOH), United Arab Emirates, and the Institutional Review Board (IRB) of Gulf Medical University. The MOH approval reference number is MOHAP/DXB‐REC/A.S.O/No. 153/2024. All procedures involving human participants were conducted in accordance with the ethical principles outlined in the Declaration of Helsinki and the World Health Organization (WHO) guidelines. Participants were fully informed about the purpose and procedures of the study, and written informed consent was obtained prior to sample collection. Data confidentiality and anonymity were strictly maintained throughout the research process.

### 2.5 Assessment of Oxidative Stress Status

#### 2.5.1 Lipid Peroxidation Assay

Lipid peroxidation levels in plasma samples were determined using the colorimetric Thiobarbituric Acid Reactive Substances (TBARS) assay, which quantifies malondialdehyde (MDA), a key marker of oxidative stress [14].

An MDA standard curve was generated and used to calculate MDA concentrations in participants' serum samples. 1 mM working solution was prepared from which the calibration curve was constructed in the concentration range of 125–0 μM.

Blood samples were promptly collected and processed to isolate plasma, which was kept on ice until further analysis. Each tube received 100 μL of plasma and 100 μL of 12% SDS lysis solution, followed by gentle vortexing and a 5‐min incubation at room temperature. Next, 1 mL of TBA reagent was added, and the tubes were sealed and mixed thoroughly before being incubated at 95°C for 45 to 60 min to form the MDA–TBA adduct. After cooling on ice for 5 min, the samples were centrifuged at 3000 rpm for 15 min, and the supernatant was transferred to cuvettes for analysis. The absorbance was measured at 532 nm using a UV–Vis spectrophotometer within 30 min to ensure stability. The MDA concentration in each sample was calculated by using the following linear regression equation obtained from the standard curve.

#### 2.5.2 Total Antioxidant Capacity (TAC)

The TAC of plasma samples was determined using the ABTS [2,2′‐azino‐bis(3‐ethylbenzothiazoline‐6‐sulfonic acid)] radical cation decolorization assay. The method quantifies antioxidant activity by monitoring the reduction of the ABTS^+^• radical, with results expressed as Trolox equivalents [15]. 100 μL of plasma sample was added to 450 μL of 10 mM phosphate‐buffered saline (PBS) + 100 μL of ABTS radical solution (300 μM) + 250 μL of 4.5 μM myoglobin and 100 μL of 250 μM H_2_O_2_. The reaction mixtures were incubated at room temperature for exactly 3 min, after which the absorbance was measured at 600 nm using a UV–Vis spectrophotometer. Serum total antioxidant capacity in the samples was calculated as Trolox equivalent (TE), using the Trolox standard curve.

### 2.6 Gene Expression Analysis of *PXDN* and *NF‐κB*

#### 2.6.1 RNA Extraction and Reverse Transcription

Total RNA was extracted from whole blood samples using the PureLink Total RNA Blood Purification Kit, Invitrogen life technologies (Cat. no. K1560‐01) and quantified using a NanoDrop spectrophotometer (Thermo Scientific) to assess purity and concentration. The RNA extracted was reverse‐transcribed into complementary DNA (cDNA) using the High‐Capacity cDNA Reverse Transcription Kit (Applied Biosystems, Thermo Fisher Scientific, Cat. No. 4368813), following the manufacturer's instructions. The resulting cDNA was stored at −20°C until further use in quantitative PCR.

#### 2.6.2 qRT‐PCR

Quantitative real‐time polymerase chain reaction was performed using the SYBR Green Master Mix (Thermo Fisher Scientific, USA) on the Applied Biosystems StepOnePlus Real‐Time PCR System, following the manufacturer's recommendations. Target‐specific primer assays for *PXDN* and *NF‐κB* genes were purchased from AI Genome international, UAE.

The comparative Ct (ΔΔCt) method was used to analyse relative gene expression. Ct values of *PXDN* and *NF‐κB* were normalized against the housekeeping gene *GAPDH* to obtain the ΔCt. The ΔΔCt was then calculated. The 2^−△△Ct^ method [16] was conducted for the analysis and measurement of relative gene expression levels, and the fold change was calculated.

## 3 Results

### 3.1 Demographic and Laboratory Findings

A total of 60 participants were recruited for this study: 40 patients with T2DM (20 patients without nephropathy and 20 patients with DN). In addition, 20 matched healthy controls. Comparing demographic data and laboratory findings between the diseased groups revealed no significant differences in age or gender. Renal impairment was evident in the lab results of patients with DN, which were significantly different from those of T2DM without nephropathy. This was evidenced by significantly lower eGFR (*p* = 0.023), microalbuminuria (*p* < 0.0001), and increased urinary microalbumin‐to‐creatinine ratio (urinary M/C ratio), with *p* < 0.0001 (Table 1).

**TABLE 1:** Demographic and laboratory tests for patient groups.

Clinical data	Diabetic (*N* = 20)	Diabetic nephropathy (*N* = 20)	*p*
Demographic data			
Age (years)	51.05 ± 11.29	56.45 ± 10.80	0.605
Gender			
Female Male	6 (30%) 14 (70%)	5 (25%) 15 (75%)	0.72^a^
Laboratory test			
HbA1c %	7.58 ± 2.15	8.25 ± 2.18	0.41
eGFR (ml/min/1.73 m2)	110.01 ± 29.0	45.05 ± 15.26	**0.023** *
Microalbuminuria (mg/L)	**10.08** (1.05)	**246.50** (759.73)	**< 0.0001** *
Urinary M/C ratio (mg/g)	**12.39** (1.87)	**455.25** (1322.23)	**< 0.0001** *

*Note:* Data shown as mean ± SD. Independent student *t*‐test is used. for parametric data.

* Bold values indicates the Significant of *p* ≤ 0.05.

^a^ Chi square test is used, gender shown as *N*(%). HbA1c %: Haemoglobin A1c (Glycated Haemoglobin), eGFR: Estimated Glomerular Filtration Rate, Urinary M/C ratio: Urinary Microalbumin‐to‐creatinine ratio. For non‐parametric data, median and interquartile range(IQR) are used. Mann–Whitney Mann–Whitney Test is used.

### 3.2 Oxidative Stress and TAC Status Among the Study Groups

**FIGURE 1:**
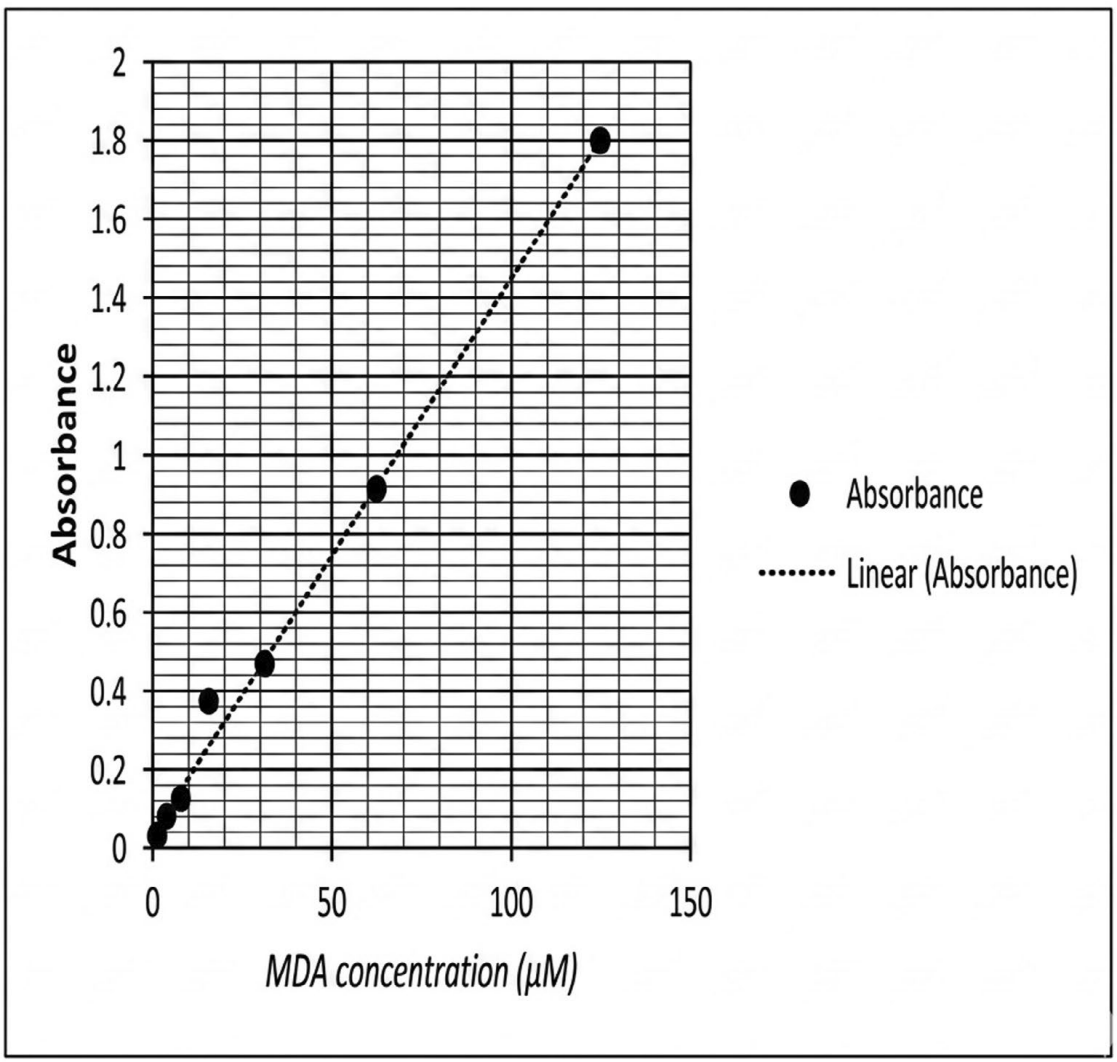
MDA standard curve. The values which are presented on the X‐axis represent MDA concentrations (μM) plotted against absorbance at 532 nm on the Y‐axis. All values were corrected for background absorbance using blank controls and reported as μM of MDA equivalents.

Linear regression analysis of the standard MDA curve. Figure 1 revealed a correlation coefficient of 0.996. The equation used in calculating the MDA concentration in participants' samples was as follows: y = 0.0145 × x + 0.0165, where.

y = represents Absorbance.

x = MDA Concentration (μM).

**FIGURE 2:**
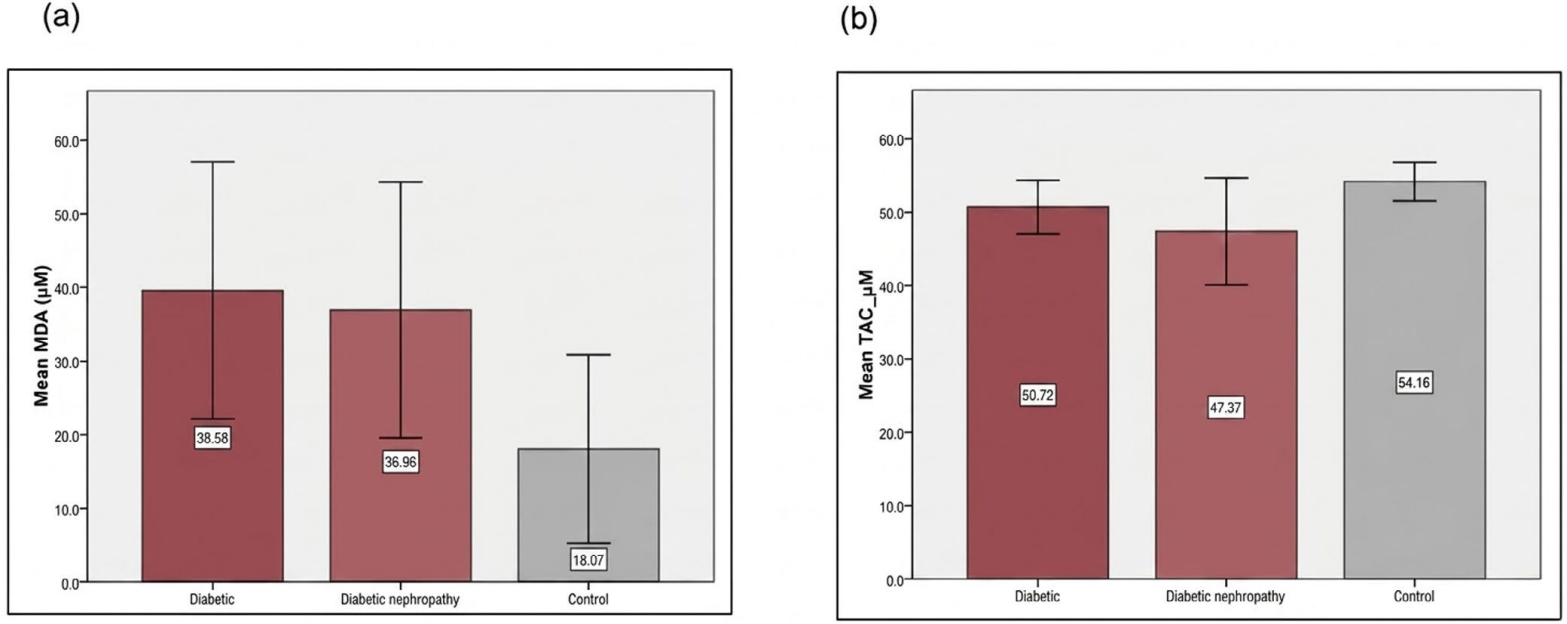
Oxidative stress among the studied groups. (a) comparison of the concentration level of MDA between the studied groups, (b) comparison of the TAC between the studied groups.

Patients with T2DM and DN showed elevated oxidative stress, indicated by significantly higher MDA levels than controls (*p* = 0.03 and *p* = 0.04, respectively). Total antioxidant capacity (TAC) was lowest in DN patients and differed significantly from both T2DM and control groups (*p* = 0.001), (Table 2, Figure 2).

**TABLE 2:** MDA levels and TAC status in serum of patients and control groups.

Serum biomarkers	DM (*N* = 20)	DN (*N* = 20)	Control (*N* = 20)	*p*
MDA (uM)	39.58 ± 17.43	36.96 ± 17.37	18.07 ± 12.78	0.96^a^, **0.03** ^b^, **0.04** ^c^
TAC (uM)	50.72 ± 3.61	47.37 ± 7.30	54.16 ± 2.64	**0.001** ^a^, 0.18^b^, **0.001** ^c^

*Note:* Data is shown as median (IQR).

^a^ Between diabetic and diabetic nephropathy.

^b^ Between diabetic and control.

^c^ Between diabetic nephropathy and control. Mann–Whitney Test is used.

* Bold values indicates the Significant of *p* ≤ 0.05.

### 3.3 Correlation of Oxidative Stress With Kidney Damage

**FIGURE 3:**
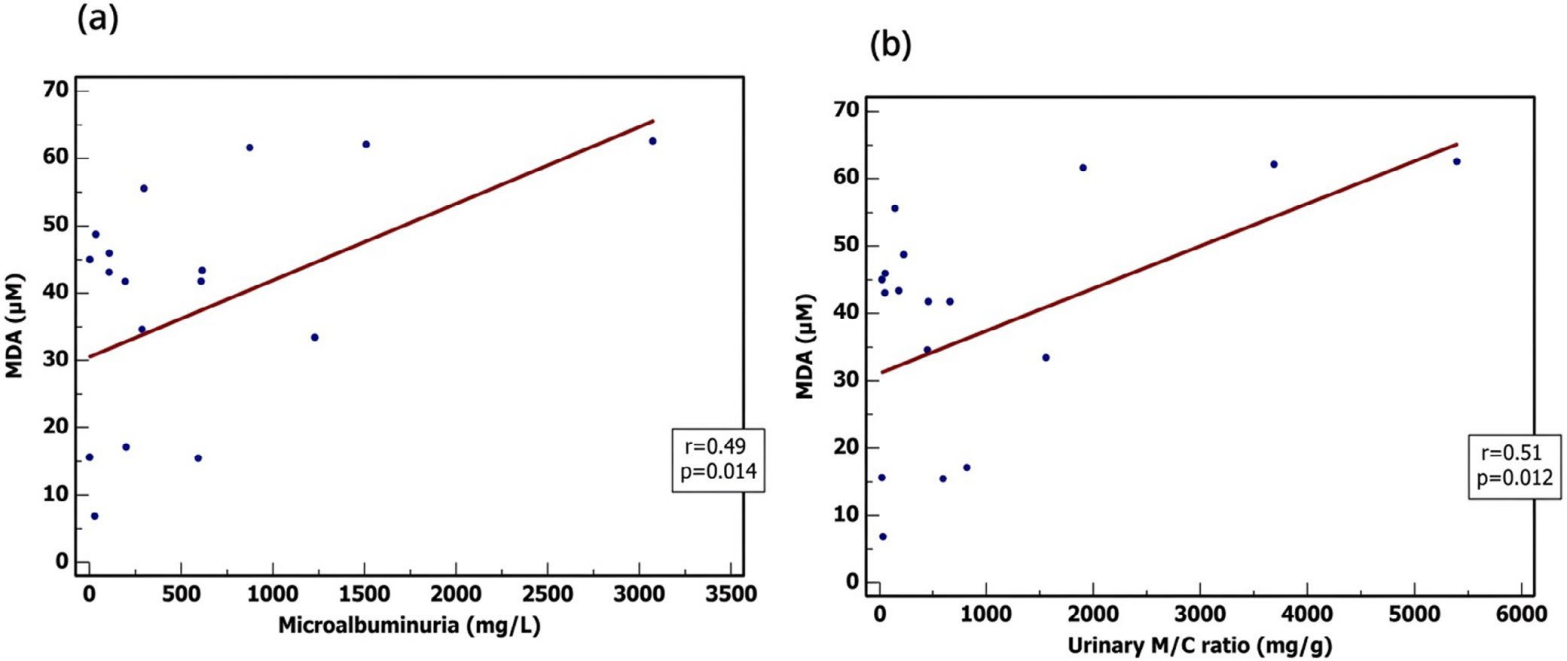
Correlations between MDA & laboratory findings in DN patients. (a) A positive correlation was detected between MDA levels and microalbuminuria, with significance; *p* = 0.014, (b) A positive correlation was detected between MDA levels and M/C ratio, with significance; *p* = 0.012. Pearson correlation test with a two‐tailed test to test the significance was used.

A statistically significant positive correlation was observed between serum MDA levels and microalbuminuria (*r* = 0.49, *p* = 0.014), indicating that higher oxidative stress is associated with increased albumin excretion. Similarly, a significant positive correlation was found between MDA and the urinary M/C ratio (*r* = 0.51, *p* = 0.012). These results suggest a direct relationship between the degree of lipid peroxidation and the extent of glomerular damage in DN patients (Figure 3).

### 3.4 *PXDN* and *NF‐κB* Gene Expression Levels in the Studied Groups

**FIGURE 4:**
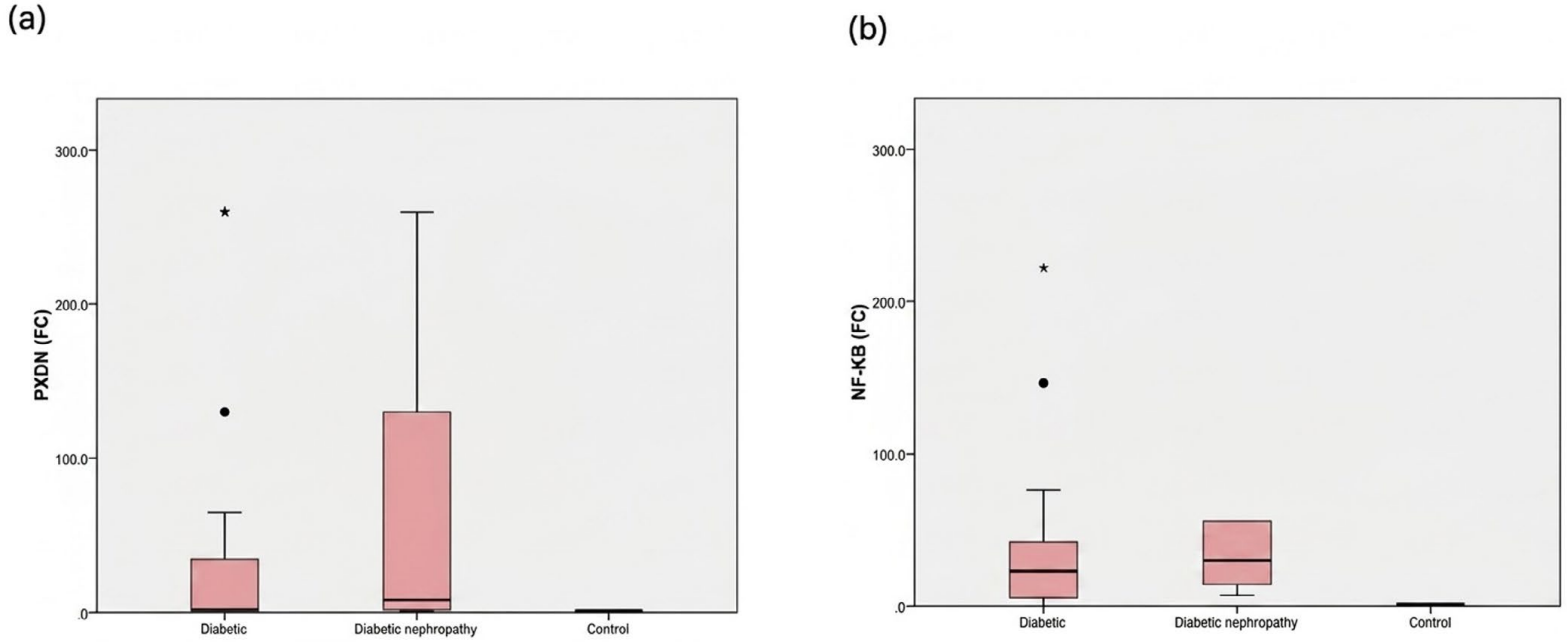
*PXDN* and *NF‐kB* expression levels among the studied groups. (a) PXDN expression in different groups, with the highest expression shown in the DN group. (b) NF‐kB expression in different groups, with the highest expression noted in the DN group. Data is shown as median fold change. The Mann–Whitney Test is used.*Significant (*p* ≤ 0.05).

The oxidative stress‐induced *PXDN* gene was upregulated in both T2DM and DN groups. Diabetic nephropathy patients exhibited a markedly higher median fold change of 10.83; four times higher than in diabetic patients (*p* = 0.023), and nearly 11 times higher than in controls (*p* < 0.0001) (Figure 4a). For *NF‐κB*, the diabetic patients showed a median fold change of 22.67, while diabetic nephropathy patients exhibited a slightly higher median of 29.76. This represents a modest increase (approximately 1.3‐fold) in diabetic nephropathy patients and a substantial increase compared to controls. Statistical analysis revealed highly significant differences between the control and diabetic groups (*p* = 0.0001) and between the control and diabetic nephropathy groups (*p* < 0.0001). However, the difference between the diabetic and diabetic nephropathy groups was not statistically significant (*p* = 0.072), (Figure 4b).

### 3.5 Diagnostic Potential of the Studied Genes for Early Detection of Diabetic Nephropathy

**FIGURE 5:**
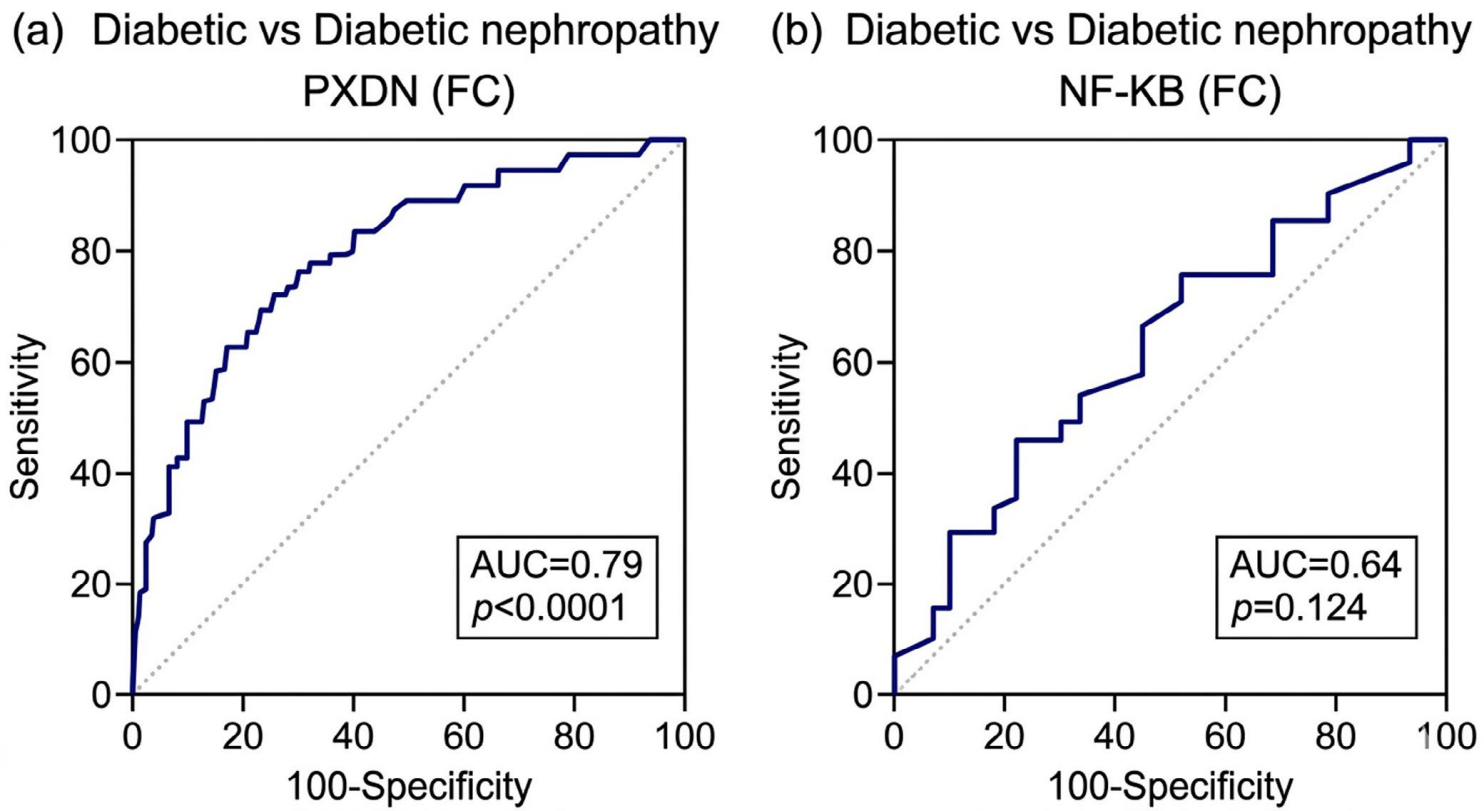
ROC analysis for the diagnostic potential of the studied genes. (a) PXDN gene ROC analysis revealed a best cut‐off value > 5.4 [sensitivity = 65%, specificity = 90%, and accuracy = 77.5%], area under the curve (AUC) = 0.79, *p* < 0.0001 (b) NF‐kB diagnostic performance showed a best cut‐off value > 3.4 [sensitivity = 99%, specificity = 25%, and accuracy = 94.15%], AUC = 0.64, and *p* = 0.124.

To evaluate the potential of *PXDN* and *NF‐kB* as diagnostic and prognostic biomarkers, Receiver Operating Characteristic (ROC) curve analyses were conducted. It was demonstrated that *PXDN* expression patterns can differentiate between T2DM and DN, at a cut‐off > 5.4, 65% sensitivity, 90% specificity, area under the curve (AUC) 0.79, and *p* < 0.0001. As for *NF‐kB*, the ROC curve revealed a cut‐off > 3.4, sensitivity 99%, very low specificity 25%, AUC 0.64, and *p* value was not significant (Figure 5).

## 4 Discussion

*PXDN* is a heme peroxidase that maintains basement membrane stability, especially in the kidneys [17]. In diabetic nephropathy, *PXDN* switches to a pathological role [6]. There is a lack of detailed studies investigating *PXDN's* regulation and role in diabetic kidney disease. This study aimed to investigate the expression of oxidative stress‐induced genes and their potential for early detection of diabetic nephropathy. Our focus was to analyse the *PXDN* gene in individuals with T2DM and to compare its expression with the pro‐inflammatory transcription factor *NF‐Κb*, a main contributor to the pathogenesis of DN.

Serum levels of MDA and total antioxidant capacity were measured in healthy controls, patients with type 2 DM without kidney disease, and diabetic patients with nephropathy to assess systemic oxidative stress status. Diabetic nephropathy patients showed significantly higher MDA and lower TAC compared to controls, indicating elevated systemic oxidative stress. These results align with prior experiments [18], including a case–control study [19], which demonstrated that oxidative imbalance is most severe in DN patients compared to both diabetics without nephropathy and healthy individuals.

Using quantitative RT‐PCR, we evaluated *PXDN* expression levels in patients with and without DN, and healthy controls. *PXDN* was upregulated in patients with DN, with levels notably higher than both healthy controls and T2DM patients without nephropathy.

These results are consistent with previous experimental studies. Zhong et al. [11] demonstrated that *PXDN* is upregulated in diabetic mouse kidneys, confirmed via qRT‐PCR, ELISA, and immunohistochemistry (IHC). In that study, *PXDN* also emerged as a top diagnostic gene from transcriptomic analysis of kidney biopsy samples using LASSO and SVM‐RFE machine learning models.

Importantly, our use of peripheral blood for *PXDN* quantification highlights its translational potential. While biopsy data provide tissue‐specific resolution, they are clinically limited. Demonstrating elevated *PXDN* in the bloodstream enhances the feasibility of its use as a non‐invasive marker for early screening or progression monitoring in T2DM patients.

Using a knockout mouse model, researchers found functional evidence for *PXDN's* role in fibrosis by showing that *PXDN*‐knockout mice develop significantly less renal fibrosis and collagen accumulation following injury [20]. Collectively, these findings indicate that *PXDN* is not merely elevated in fibrotic kidneys but is likely contributing to the progression of tissue remodelling.

Previous studies suggested that it may also be modulated by redox signalling. Another study [21], for instance, identified *PXDN* as a redox‐responsive gene in prostate cancer, where its expression increases under oxidative stress conditions. Several studies have highlighted oxidative stress as a key factor in the progression of diabetic kidney disease, including its effects on glomerular and tubular compartments [4, 22]. Cao et al. [23] similarly found that *PXDN* promotes hypochlorous acid (HOCl) production in endothelial cells through NOX2, contributing to oxidative vascular injury.

*PXDN* expression in our study did not significantly correlate with either MDA or TAC levels. This finding indicates that *PXDN* expression level is not part of the broad oxidative stress–responsive gene set often triggered by global redox imbalance. Instead, *PXDN* may respond to localized oxidative stress or fibrosis‐linked intracellular signalling that is not captured by serum markers like MDA or TAC. Clinically, this distinction could improve biomarker specificity. Whereas systemic markers can be influenced by diet, infection, or transient inflammation, *PXDN's* expression profile may reflect more stable, disease‐related remodelling processes within the kidney.

We also investigated the expression of *NF‐κB*, a transcription factor classically activated by both oxidative stress and inflammation. In diabetic complications, *NF‐κB* is often upregulated and drives expression of cytokines and adhesion molecules. While *NF‐κB* levels were increased in DN patients, no correlation was found between *NF‐κB* and *PXDN* expression. One study found that *NF‐κB* expression closely tracked with pro‐inflammatory cytokines (IL‐6, IL‐1β, TNF‐α), reactive oxygen species production, and antioxidant enzyme dysregulation (e.g., SOD, HO‐1, CAT) [24]. These findings support the view that *NF‐κB* may not directly regulate ECM structural components such as *PXDN*, despite its involvement in oxidative stress pathways.

ROC analysis was performed to evaluate the diagnostic potential of the studied genes. At a cut‐off value of > 5.4, *PXDN* achieved a sensitivity of 65% and a specificity of 90%, with an overall diagnostic accuracy of 77.5%. Although the sensitivity was moderate, the high specificity indicated a strong ability to identify patients with nephropathy. The ROC curve constructed for *NF‐kB*, revealed a diagnostic potential for DN at a cut‐off > 3.4, with a sensitivity 99%, a very low specificity 25%, AUC 0.64, and *p* value was not significant. These findings underscore the potential of *PXDN* not only as a diagnostic marker but also as a prognostic tool for detecting progression from diabetes to diabetic nephropathy, reinforcing its clinical value in monitoring disease advancement.

Our results are further supported by Makhanya [25], who showed that *PXDN* expression increases in fibrotic tissues independently of canonical inflammatory pathways. *PXDN* may thus belong to a regulatory niche separate from typical *NF‐κB*–driven inflammation, potentially regulated by factors such as SMADs or EGR1, particularly under TGF‐β1 stimulation, which was experimentally shown to drive *PXDN* expression in fibrotic human epithelial models.

A study using renal tissue from mice [9] reported that *PXDN* is detectable in both healthy and diseased kidneys but adopts a more active, ECM‐remodelling role under prolonged fibrotic or oxidative stress. In this sense, *PXDN* resembles other matrix‐related enzymes like *LOX*, which normally perform important physiological functions but can become harmful when chronically overexpressed. For example, it was reported [26], that *LOX* expression increases in diabetic kidneys, particularly in myofibroblasts during fibrosis, and that inhibiting *LOX* reduced fibrosis and improved kidney function. Similarly, Zhang et al. [27] found that *LOX* levels in both serum and kidney tissue correlate strongly with fibrosis severity in chronic kidney disease patients, indicating *LOX* as a potential biomarker for renal damage. These findings illustrate how enzymes such as *LOX*, like *PXDN*, contribute to pathological remodelling in kidney disease.

## 5 Limitations

- The cross‐sectional design of this study limits the ability to conclude causality. Although *PXDN* levels were found to be elevated in individuals with diabetic nephropathy, it remains unclear whether this upregulation precedes the onset of kidney damage or occurs as a consequence of disease progression. Longitudinal studies are necessary to determine whether changes in *PXDN* expression emerge during the early stages of nephropathy and whether this gene could serve as a reliable early indicator of disease development.
- Peripheral blood sampling does not provide tissue‐specific resolution. The origin of *PXDN* mRNA detected in circulation cannot be definitively attributed to renal tissue without corresponding biopsy data.
- The sample size in this study, although sufficient for statistical comparisons, was relatively small and drawn from a single‐center population. This may limit the generalizability of the findings to broader clinical contexts, including patients of different ethnicities, geographical backgrounds, or comorbid conditions. Variability in *PXDN* expression and oxidative stress responses across diverse populations cannot be excluded. Larger, multi‐center studies are needed to validate *PXDN* as a potential biomarker for diabetic nephropathy and to assess its performance across different clinical subgroups.

## 6 Conclusion

This study shows that PXDN expression is significantly higher in patients with diabetic nephropathy than in healthy controls and diabetic patients without nephropathy, implying a role in kidney‐specific pathological remodelling. Although systemic oxidative stress markers (MDA and TAC) were altered in nephropathy patients, PXDN expression did not correlate with these markers or with NF‐κB levels, suggesting it is not governed by generalized oxidative stress or canonical inflammatory pathways. Overall, the results indicate PXDN is selectively induced under renal stress in diabetes, supporting its potential as a disease‐specific biomarker and warranting further investigation into its tissue‐level regulatory mechanisms.

## Author Contributions

**Aisha Anees Ahmed:** investigation, methodology, data curation, writing – original draft, funding acquisition, formal analysis. **Mahir Khalil Jallo:** investigation, resources, writing – review and editing, methodology, validation, data curation, formal analysis. **Dalia A. Gaber:** conceptualization, methodology, investigation, writing – original draft, funding acquisition, formal analysis. **Zakira Hanin:** methodology, investigation, data curation, writing – original draft, funding acquisition, formal analysis. **Shuhd Salem Al Nahdi:** methodology, data curation, investigation, writing – original draft, funding acquisition, formal analysis. **Sumayya Althaf Kasim:** methodology, data curation, investigation, writing – original draft, funding acquisition, formal analysis. **Rajaram Rhambau Jagdale:** investigation, methodology, validation, formal analysis, data curation.

## Funding

This research received a grant from Gulf Medical University, UAE.

## Ethics Statement

This study was conducted following strict ethical standards to ensure the protection of participant rights and confidentiality. Ethical approval for the study was obtained from both the Ministry of Health and Prevention (MOH), United Arab Emirates, and the Institutional Review Board (IRB) of Gulf Medical University. The MOH approval reference number is MOHAP/DXB‐REC/A.S.O/No. 153/2024. All procedures involving human participants were conducted in accordance with the ethical principles outlined in the Declaration of Helsinki and the World Health Organization (WHO) guidelines.

## Consent

Participants were fully informed about the purpose and procedures of the study, and written informed consent was obtained prior to sample collection. Data confidentiality and anonymity were strictly maintained throughout the research process.

## Conflicts of Interest

The authors declare no conflicts of interest.

## Data Availability

The data that support the findings of this study are available on request from the corresponding author. The data are not publicly available due to privacy or ethical restrictions.
